# Tumour Suppressor Adenomatous Polyposis Coli (APC) localisation is regulated by both Kinesin-1 and Kinesin-2

**DOI:** 10.1038/srep27456

**Published:** 2016-06-07

**Authors:** Peter T. Ruane, Laura F. Gumy, Becky Bola, Beverley Anderson, Marcin J. Wozniak, Casper C. Hoogenraad, Victoria J. Allan

**Affiliations:** 1 Faculty of Life Sciences, University of Manchester, Oxford Road, Manchester, M13 9PT, UK; 2 Division of Cell Biology, Faculty of Science, University of Utrecht, Padualaan 8, 3584CH, Utrecht, The Netherlands

## Abstract

Microtubules and their associated proteins (MAPs) underpin the polarity of specialised cells. Adenomatous polyposis coli (APC) is one such MAP with a multifunctional agenda that requires precise intracellular localisations. Although APC has been found to associate with kinesin-2 subfamily members, the exact mechanism for the peripheral localization of APC remains unclear. Here we show that the heavy chain of kinesin-1 directly interacts with the APC C-terminus, contributing to the peripheral localisation of APC in fibroblasts. In rat hippocampal neurons the kinesin-1 binding domain of APC is required for its axon tip enrichment. Moreover, we demonstrate that APC requires interactions with both kinesin-2 and kinesin-1 for this localisation. Underlining the importance of the kinesin-1 association, neurons expressing APC lacking kinesin-1-binding domain have shorter axons. The identification of this novel kinesin-1-APC interaction highlights the complexity and significance of APC localisation in neurons.

Neurons are highly polarised cells which require the structural and organisational capacity of the microtubule (MT) cytoskeleton and its associated proteins (MAPs)^1^. By regulating MT interactions and dynamics, the coordinated activities of a variety MAPs tailor the MT cytoskeleton to specific functions^2^. In neurons, stable MTs bundle in the axon to serve as both a structural framework and a directional highway for the transport of materials by motor proteins. Conversely, the dynamics of MT growth and shrinkage at the axon tip provide the plasticity to steer axon growth and alter synaptic activity^1^.

One MAP intimately involved with cell polarity is Adenomatous Polyposis Coli (APC)^3^. APC functions through interactions with MTs, the MT plus end-interacting (+TIP) EB proteins, microtubule-based kinesin motors and both the actin and intermediate filament cytoskeletal elements^4^,^5^,^6^,^7^,^8^,^9^,^10^,^11^. As part of the discrete β-catenin destruction complex, APC also acts in the wnt signalling pathway with GSK3β, Axin and CK1ε^12^,^13^. A combination of modular tertiary structure and extended unstructured regions provides the platform for these myriad interactions across the 2843-residue APC protein^14^. Partial loss of these interactions after nonsense mutations mark APC as a tumour suppressor associated with both syndromic and spontaneous tumourigenesis^15^,^16^,^17^. Highlighting important roles in the central nervous system, deletion of APC is also linked with intellectual and autistic disorders^18^.

APC is important for brain development and functions in pathways controlling neuronal differentiation^19^,^20^,^21^. Initially found at the tips of multiple neurites, APC becomes localised specifically at the distal region of the growing axon, termed the growth cone, upon neuronal polarisation, perhaps in response to local inhibition of GSK3β^13^,^22^,^23^,^24^,^25^,^26^,^27^,^28^,^29^. At this site APC plays important roles in axonal growth, guidance, and morphology by promoting MT growth and stabilisation, actin remodelling and the translation of localised RNAs^28^,^30^,^31^,^32^,^33^,^34^.

The accumulation of APC at the axon tip is an important but incompletely understood aspect of its function. Around half of the 45 kinesin superfamily (KIF) members are MT plus end-directed processive motors, many of which act to traffic cargo along axons^35^. Kinesin-2 motors have been suggested to play a role in transport of APC into the axon^26^. The KAP3 subunit of heterotrimeric kinesin-2 and the homodimeric kinesin-2 KIF17 bind the N-terminus of APC to transport it to the cell periphery in fibroblasts and epithelial cells^5^,^6^. Whilst heterotrimeric kinesin-2 is an axonal motor^36^, KIF17 is an unlikely candidate to localise APC to the axon since it transports cargo selectively to dendrites^37^,^38^. In addition, EB proteins associate with the C-terminus of APC, potentially enabling APC to track the growing plus end of MTs at the axon tip^4^,^31^,^33^.

Intriguingly, a genetic interaction between APC and kinesin-1 in Drosophila impacts upon polarised membrane trafficking in neurons^39^. Moreover, in mouse epithelial cells kinesin-1 has been reported to localise APC to the epithelial cell periphery^40^. Kinesin-1 is the prototypic KIF member^41^, and was subsequently characterised as a key axonal cargo transporter that also accumulates in axonal growth cones^42^. Kinesin-1 can function as a homodimer of MT motor domain-containing kinesin heavy chain subunits (KIF5), with kinesin light chain (KLC) proteins able to associate with KIF5 to provide further cargo-binding and autoregulatory functions^43^.

In the present study, an interaction between KIF5 and the C-terminus of APC was identified which contributes to the peripheral localisation of GFP-APC in human fibroblasts. In rat hippocampal neurons GFP-APC enrichment at the axon tip was drastically reduced in a mutant GFP-APC lacking the KIF5-binding domain. Interestingly, expression of this mutant led to shorter axons without affecting growth cone morphology. In addition, inhibiting kinesin-1 activity by expression of dominant negative (DN) constructs prevented APC accumulation at axon tips. Moreover, heterotrimeric kinesin-2 DN constructs also suppressed axon tip localisation of APC. These data suggest that kinesin-1 and kinesin-2 collaborate to localise APC to the axon tip and that the interaction of APC with kinesin-1 is required for proper axon growth.

## Results

### KIF5 directly binds a novel region in the APC C-terminus

**Figure 1:**
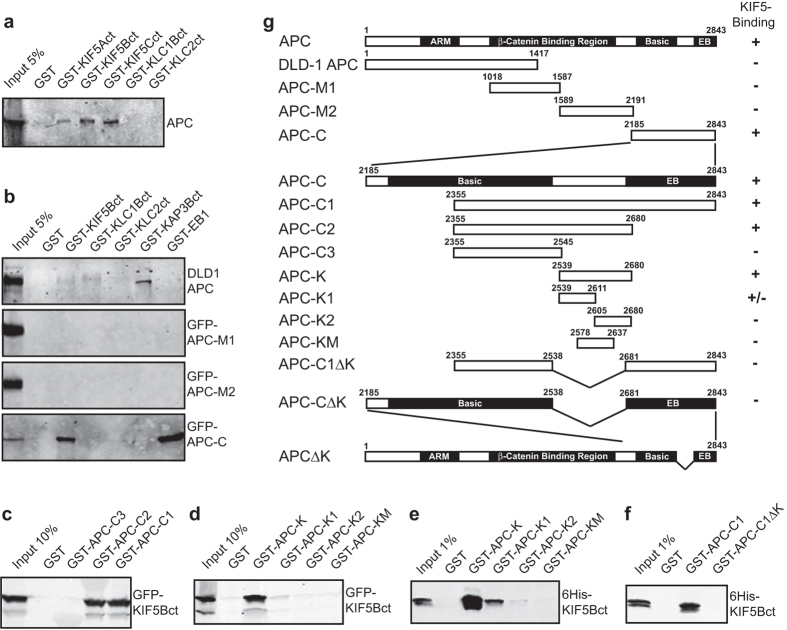
KIF5 kinesin-1 heavy chains directly bind the C-terminus of APC. (**a**) Bacterially expressed GST-tagged kinesin-1 constructs were incubated with MRC-5 cell lysate, pulled down with glutathione beads and immunoblotted with anti-APC. (**b**) GST-tagged kinesin-1, kinesin-2 or EB1 proteins were incubated with DLD-1 cell lysate or with transfected HeLaM cell lysate and pulled down using glutathione beads. HeLaM cells were transfected with APC mid region fragments (GFP-APC-M1 or GFP-APC-M2) or APC C-terminus (GFP-APC-C). Pull downs from DLD-1 lysate were immunoblotted using anti-APC and pull downs from transfected HeLaM lysates were immunoblotted with anti-GFP. (**c**,**d**) GST-tagged C-terminal APC proteins were used in pull downs of GFP-KIF5Bct-transfected HeLaM lysates. (**e**,**f**) GST-tagged C-terminal APC proteins were used in pull downs with bacterially expressed 6His-KIF5Bct and immunoblots were performed with anti-6His. (**g**) A schematic showing full length APC, the truncated APC endogenously expressed in DLD-1 cells and the APC constructs used in this work. Functional domains in APC are shaded and labelled: ARM, Armadillo repeats; Basic, basic MT-binding domain; EB, EB protein-binding region. Amino acid numbers are displayed. KIF5 binding capability is shown, summarised from data in this figure. APC constructs were N-terminally tagged with GFP for expression in mammalian cells or with GST for bacterial expression. Images of the Ponceau-S-stained membranes for all panels are shown in Supplemental Fig. 1.

Since links between kinesin-1 and APC have been reported^39^,^40^, we tested for a biochemical interaction between APC and kinesin-1. Kinesin-1 functions as a KIF5 dimer or a tetramer of KIF5 plus two KLCs that associate with the KIF5 C-terminus. Kinesin-1 couples to cargo either via the central stalk and C-terminal tail domains of KIF5, and/or through binding between cargo and KLCs^42^. We expressed the C-terminal cargo-binding tails of human KIF5A, B and C as GST-fusion proteins, and found that all were able to pull down endogenous APC from MRC-5 human fibroblast lysates (Fig. 1a). In contrast, the C-terminal cargo-binding region of human KLC isoforms KLC1B and KLC2 expressed as GST fusions did not interact with APC (Fig. 1a).

To begin to map the portion of APC that interacts with kinesin-1, the truncated APC endogenously expressed in the DLD-1 colon cancer cell line was used (truncated at amino acid 1417, Fig. 1g). This form lacks the C-terminal EB1 binding site but contains the N-terminal armadillo domains that bind the non-motor subunit of heterotrimeric kinesin-2, KAP3 (refs 4 and 6; Fig. 1b). As expected, a C-terminal fragment of KAP3B containing the known APC-interacting region^6^, GST-KAP3Bct, pulled down DLD-1 APC whereas GST-EB1 did not (Fig. 1b). GST-KIF5Bct did not interact with DLD-1 APC, suggesting the C-terminal half of APC associates with kinesin-1.

We dissected the kinesin-1 interaction site further by expressing GFP-tagged C-terminal APC fragments (Fig. 1g) in HeLaM cells and testing their ability to interact with recombinant GST-tagged KIF5Bct, KAP3Bct and EB1. KAP3Bct did not interact with any of these GFP-APC proteins, as expected (Fig. 1b). In contrast, EB1 and KIF5Bct both pulled down the C-terminal third of APC (amino acids 2185–2843), but not the APC central sections (Fig. 1b). Further delineation of the kinesin-1 binding site to residues 2539–2680 of APC (APC-K) was achieved using bacterially expressed GST-APC fragments to pull down GFP-KIF5Bct from HeLaM cell lysates (Fig. 1c,d). Interestingly, this region of APC lies between the basic MT-binding site and the EB protein binding-SxIP motif, and has not been specifically mapped as a protein interaction site^14^. The kinesin-APC interaction is direct, since purified 6His-KIF5Bct was pulled down by GST-APC-K (Fig. 1e). Further subdivision of the K domain (APC-K1, -K2 and -KM, Fig. 1g) greatly reduced the interaction with GFP-KIF5Bct from cell lysates (Fig. 1d), or with purified KIF5Bct (Fig. 1e), although APC-K1 displayed a low level of binding. Confirming that amino acids 2539–2680 were needed for kinesin-1 binding, an APC construct lacking this region, GST-APC-C1ΔK, did not pull down 6His-KIF5Bct (Fig. 1f).

**Figure 2:**
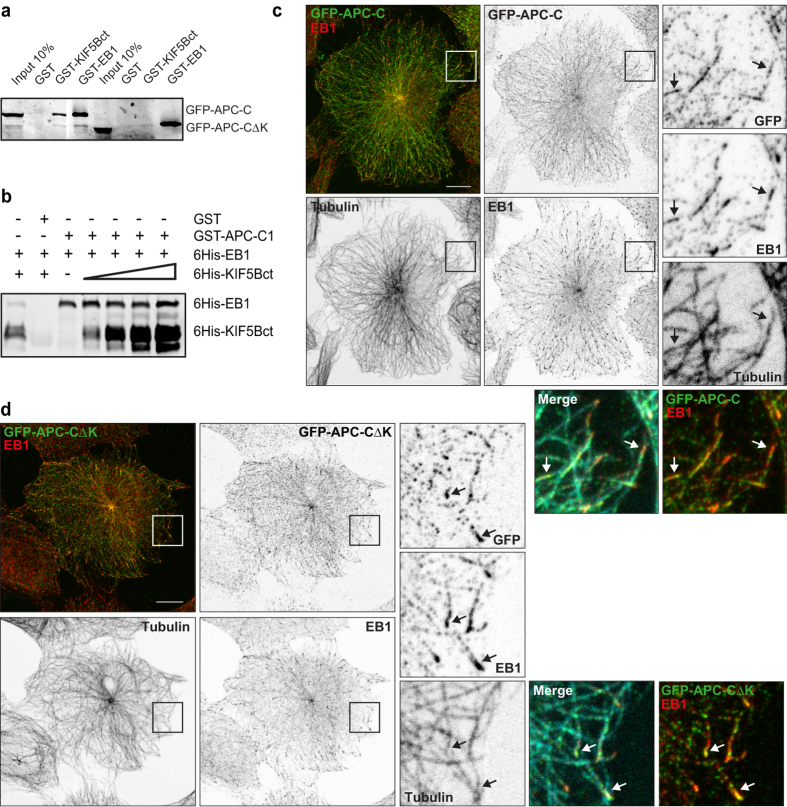
The KIF5 binding domain of APC is not needed for interaction of the APC C-teriminal domain with EB1 or microtubule plus tips. (**a**) Purified GST-KIF5Bct or GST-EB1 were incubated with HeLaM cell lysate after transfection with GFP-APC-C or GFP-APC-CΔK. Pull downs were immunoblotted with anti-GFP. (**b**) GST-APC-C1 was used to pull down 6His-EB1 in the presence of increasing amounts of 6His-KIF5Bct. Immunoblotting with anti-6His antibody revealed no competition for binding between 6His-EB1 and 6His-KIF5Bct. Supplemental Fig. 2 shows the Ponceau-S staining of the membranes used in panels (**a**,**b**). (**c**,**d**) MRC-5 cells were transfected with GFP-APC-C (**c**) or GFP-APC-CΔK (**d**), fixed and labelled with anti-tyrosinated-α-tubulin, anti-EB1 and anti-GFP antibodies. Cells were imaged by confocal microscopy. Z-series maximum projections of all image planes are shown in the main panels. Single channels are shown in reverse contrast, along with a two colour merge of the GFP and EB1 channels. For enlarged areas, three z-planes were projected. Merges of all three labels and two colour overlays of APC and EB1 are shown for the enlargments. GFP-APC-C and GFP-APC-CΔK both localise to a subset of MT ends, where EB1 is also enriched (arrows). Scale bars = 10 μm; enlarged regions are 10 × 10 μm.

To investigate how the APC-K region affects APC localisation and interactions in cells, we generated a GFP-tagged APC C-terminal construct that contained the MT and EB binding domains, with or without the kinesin-1 interaction domain (GFP-APC-C and GFP-APC-CΔK, respectively). As expected, GFP-APC-CΔK was not pulled down from HeLaM lysates by GST-KIF5Bct, although it interacted strongly with GST-EB1 (Fig. 2a). Furthermore, biochemical examination demonstrated that KIF5 and EB1 interact independently with the APC C-terminus, since increasing 6His-KIF5Bct concentrations did not inhibit 6His-EB1 binding to GST-APC-C1 (Fig. 2b). To test the significance of this interaction for APC localisation in cells, we expressed GFP-tagged constructs in MRC5 cells, which are flat fibroblasts with a sparse, centrosome-focussed microtubule network^44^, unlike HeLaM cells. As previously described, the APC C-terminus associates with MTs but does not accumulate in peripheral clusters^11^,^45^,^46^,^47^. The KIF5-binding region was not needed for MT localisation, nor for the overlap of APC-C with EB1 at the ends of a subset of microtubules (Fig. 2c,d, arrows). Expression of high levels of truncated APC-C caused redistribution of EB1 along the length of APC-positive MTs, and this was not affected by the removal of the KIF5 binding domain (data not shown).

### Kinesin-1 promotes GFP-APC peripheral localisation in fibroblasts through the interaction with APC C-terminus

**Figure 3:**
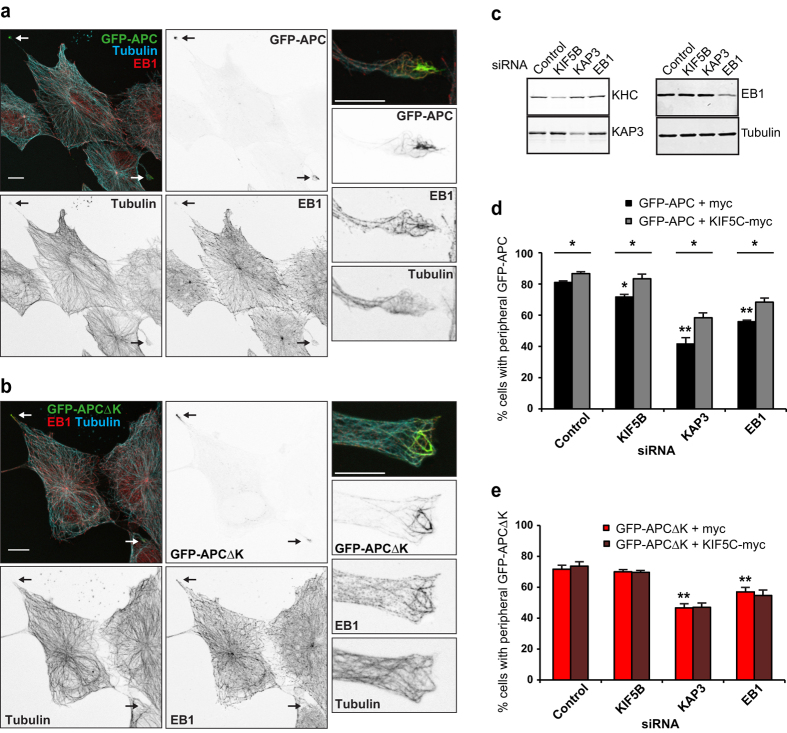
Kinesin-1 contributes to the localisation of full length APC in MRC-5 fibroblasts through an interaction with the APC C-terminus. (**a**,**b**) MRC-5 cells transfected with full length GFP-tagged APC with (**a**: GFP-APC) or without (**b**: GFP-APCΔK) the KIF5 binding region were labelled with anti-tyrosinated-α-tubulin (cyan) and anti-EB1 (red). The low and high magnification views are from different cells. GFP-APC and GFP-APCΔK both localise to distinct peripheral regions (arrows), forming clusters and aligning with MTs and EB1. Scale bars 10 μm. (**c**) MRC-5 cells were transfected with siRNAs targeted to KIF5B, KAP3 and EB1, or a control siRNA, and incubated for 72 hours. Lysates were prepared and immunoblotted with KHC, KAP3, and EB1 antibodies, alongside anti-α-tubulin as a loading control. (**d**,**e**) MRC-5 cells were transfected with siRNAs, incubated for 48 hours before cotransfection with GFP-APC or GFP-APCΔK, and either empty myc vector or KIF5C-myc/his, then incubated for a further 24 hours. Immunostaining using anti-GFP was performed and the number of cells with peripherally localised GFP-APC or GFP-APCΔK was scored. Means +/− SEM from 3 independent experiments, n = 150 cells scored per condition. Not significant (NS), *p < 0.05, **p < 0.01. Significance was determined by two-way ANOVA for comparison of control siRNA with targeted siRNA treatments, both cotransfected with GFP-APC constructs and empty myc vector. Independent sample t-tests were used for pairwise comparison of empty myc vector with KIF5C-myc/his transfectants for each siRNA condition. In addition, an independent sample t-test demonstrated p < 0.05 significance between control siRNA-treated cells expressing empty myc vector together with GFP-APC (**d**) vs. GFP-APCΔK (**e**). See Supplemental Fig. 3 for example images of cells where GFP-APC and GFP-APCΔK were not peripherally localised.

Full length APC localises to growing MT plus ends, decorates a subset of MTs at the cell periphery and forms clusters at these peripheral sites^11^,^46^,^47^,^48^,^49^,^50^, with this distribution depending on interactions with MTs, EB1 and kinesin-2 (refs 4, 5, 6). Accordingly, GFP-APC expressed in MRC-5 fibroblasts predominantly localised to a subset of MTs and in clusters at the periphery (Fig. 3a). In 15–20% of cells, APC localised to microtubules in the cell body only (Fig. S3). Full length APC lacking the kinesin-1 binding region, GFP-APCΔK, exhibited localisation that was not obviously different from GFP-APC (Fig. 3b).

Although the kinesin-1 interaction is not required *per se* for APC’s MT and +TIP binding, we set out to determine if the interaction with kinesin-1 contributed more subtly to the peripheral localisation of GFP-APC in MRC-5 fibroblast cells. Corroborating roles for heterotrimeric kinesin-2 and EB1 in APC localisation, siRNA-mediated depletion of KAP3 or EB1 (Fig. 3c) significantly reduced the proportion of MRC-5 cells exhibiting peripheral accumulations of GFP-APC by 48.7% and 31.1%, respectively (Fig. 3d, black bars). Partial knockdown of KIF5B (Fig. 3c), which is the predominant kinesin-1 family member expressed in MRC-5 cells (data not shown), led to a significant 11.5% decrease in cells with peripheral GFP-APC (Fig. 3d), suggesting that kinesin-1 does indeed facilitate peripheral APC localisation in fibroblasts. Exogenously-expressed KIF5C moves along microtubules and accumulates at the cell periphery through motor activity^51^. We therefore expressed mouse KIF5C-myc/his to test whether this would rescue the effects of KIF5B, KAP3 or EB1 depletion on APC localisation. KIF5C-myc/his co-expression indeed rescued the decrease in cells with peripheral GFP-APC after KIF5B knockdown, as well as significantly enhancing the number of control cells exhibiting peripheral GFP-APC (Fig. 3d, grey bars). Moreover, KIF5C-myc/his co-expression partially rescued the effects of KAP3 or EB1 knockdown on APC distribution (Fig. 3d), suggesting that kinesin-1 can transport APC along microtubules to the cell periphery.

We next used GFP-APCΔK to see if the direct KIF5-APC interaction lies behind these rescue data. A significantly lower number of cells exhibited peripherally localised GFP-APCΔK when compared with GFP-APC in the same experiments (11.5% reduction) (Fig. 3d,e). Furthermore, the number of cells with peripherally localised GFP-APCΔK was not affected by KIF5B depletion, nor did it increase after co-expression of KIF5C-myc/his (Fig. 3e). KIF5C-myc/his co-expression also did not rescue the decreased number of cells with peripheral GFP-APCΔK after KAP3 or EB1 knockdown (Fig. 3e). Together, these experiments demonstrate that kinesin-1 drives enrichment of APC in the cell periphery through an interaction between KIF5 C-terminal tail and residues 2539–2680 of APC.

### Deletion of the kinesin-1 binding site reduces the axon tip enrichment of APC, resulting in shorter axons

**Figure 4:**
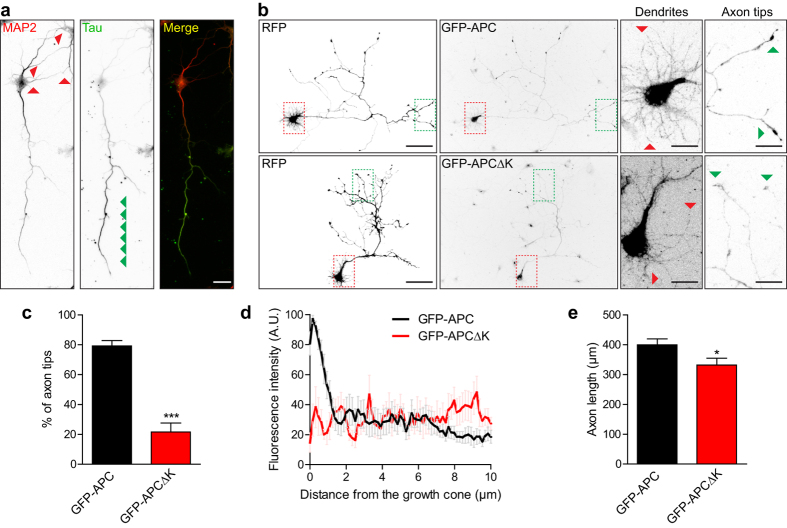
The kinesin-1 binding site is required for the enrichment of APC in the growth cone and is necessary for axonal growth. (**a**) E18 rat hippocampal neuron at 1 DIV immunostained for the dendritic marker MAP2 and the axonal marker Tau. Red arrowheads indicate short MAP2-positive dendrites whereas the green arrowheads indicate the Tau-labelled axon. Scale bar 20 μm. (**b**) E18 rat hippocampal neurons transfected at 1 DIV with GFP-APC or GFP-APCΔK constructs and fixed at 4 DIV. PFA fixation and immunostaining using anti-GFP antibody was performed. Red arrowheads indicate dendritic tips near the cell body. Green arrowheads highlight axon tips or growth cones. Scale bars 100 μm, or 20 μm in enlargements. (**C**) Quantification of the percentage of axon tips or growth cones enriched with GFP-APC or GFP-APCΔK. Shown are means  +/−  SEM from 4 independent experiments, n = 181 and n = 147 per condition, respectively. An independent sample t-test was used for comparison. ***p < 0.001. (**d**) Linescan plots showing the fluorescence intensity distribution of GFP-APC or GFP-APCΔK along the axon. Shown are means +/− SEM from 3 independent experiments, n = 12 and n = 8, respectively. (**e**) Quantification of the longest axonal neurite per neuron transfected with GFP-APC or GFP-APCΔK. Shown are means  +/− SEM from 4 independent experiments, n = 57 and n = 42 per condition, respectively. An independent sample t-test was used for comparison. *p < 0.05.

APC localises to the distal portion of the axon in neurons^24^,^26^,^27^,^28^, but the mechanism behind this localisation has not been fully elucidated. While kinesin-1 plays a subtle role in APC localisation in fibroblasts (Figs 2 and 3), we wanted to test its role in APC transport in neurons, since kinesin-1 is an important motor for axonal transport. Isolated E18 rat hippocampal neurons develop a clearly distinguishable primary neurite during day 1 *in vitro* (DIV) which is at least three times longer than the other processes and accumulates the axonal marker Tau (Fig. 4a). This very long Tau-labelled neurite is poised to become the future axon whereas the other shorter neurites develop into dendrites^28^,^52^,^53^. We transfected these neurons with full length GFP-APC or the KIF5 binding mutant GFP-APCΔK to compare their localisations. Strikingly, a highly significant reduction in the proportion of axon tips enriched for GFP-APCΔK was found when compared to GFP-APC (72.7% reduction: Fig. 4b,c). Measuring the fluorescence intensity along the distal axon demonstrated that GFP-APC was greatly enriched within the distal 1μm of the axon tip, whereas no enrichment in the axon tip over proximal regions was seen for GFP-APCΔK (Fig. 4d). These data implicate the interaction between kinesin-1 and APC in the distal axon localisation of APC in neurons.

Surprisingly, neurons expressing GFP-APCΔK had primary axons that were 16.9% shorter than neurons expressing GFP-APC (Fig. 4b,e). While shorter axons are not seen after conditional knockout of APC from mouse cortical neurons^30^, the expression of APC DN fragments and enhanced APC-β-catenin binding both lead to shorter axons in mouse dorsal root ganglion (DRG) neurons^28^,^34^. Furthermore, conditional knockout and micro-scale chromophore-assisted laser inactivation of APC both caused defects in growth cone morphology^30^,^31^. We looked in detail at growth cone morphology in neurons expressing GFP-APCΔK, but did not identify any morphological differences which might account for reduced axon growth due to growth cone defects (data not shown).

**Figure 5:**
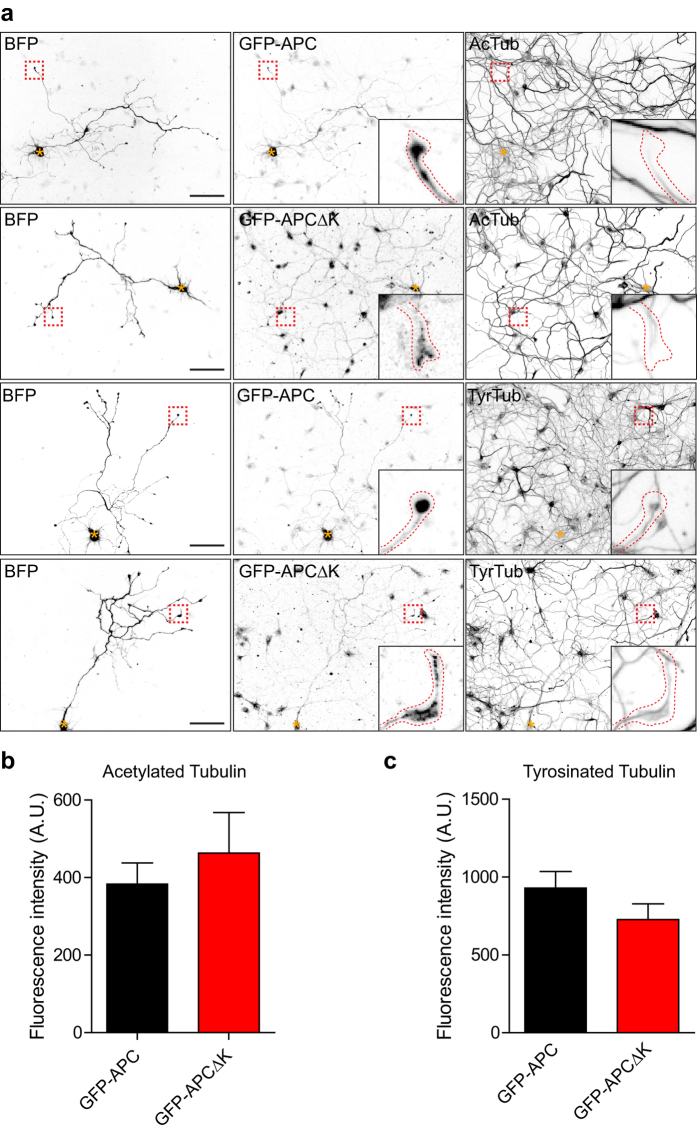
Overexpression of GFP-APC∆K does not change microtubule stability in the growth cones of hippocampal neurons. (**a**) E18 rat hippocampal neurons were cotransfected with BFP and GFP-APC or GFP-APC∆K and immunostained for acetylated tubulin (AcTub) or tyrosinated α-tubulin (TyrTub). Asterisks indicate cell bodies and insets show enlarged growth cones. Scale bar 100 μm. (**b**) Quantification of fluorescence intensity levels of acetylated tubulin in the growth cones of GFP-APC or GFP-APC∆K transfected hippocampal neurons. Shown are means  +/− SEM from 3 independent experiments, n = 25 and n = 27 neurons respectively. (**c**) Quantification of fluorescence intensity levels of tyrosinated α-tubulin in the growth cones of GFP-APC or GFP-APC∆K transfected hippocampal neurons. Shown are means  +/− SEM from 3 independent experiments, n = 23 and n = 27 neurons respectively.

Since APC interacts with microtubules, we analysed microtubule stability in the growth cones of GFP-APC and GFP-APCΔK transfected hippocampal neurons to check for possible differences which could be responsible for this reduction in axon length. Quantification of immunofluorescence levels of acetylated (stable) or tyrosinated (dynamic) microtubules showed an overall slight increase in stable microtubules in the growth cones of GFP-APCΔK transfected neurons, but these differences were not statistically significant (Fig. 5).

### Dominant negative inhibition of kinesin-1 or heterotrimeric kinesin-2 prevents axon tip localisation of APC

**Figure 6:**
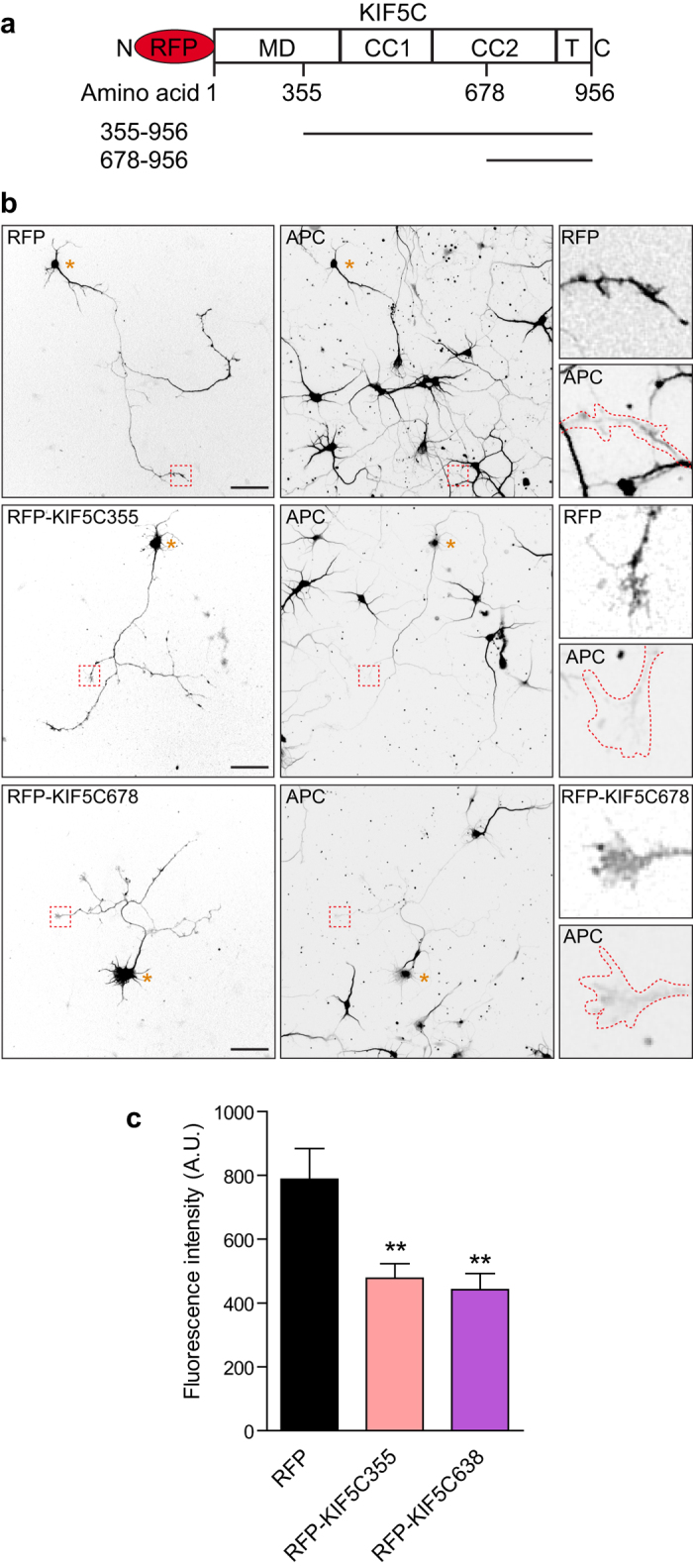
Kinesin-1 binding is required for the axonal growth cone enrichment of endogenous APC. (**a**) E18 rat hippocampal neurons transfected at 1 DIV with RFP, RFP-KIF5C355 or RFP-KIF5C678 constructs and fixed at 3 DIV. PFA fixation and immunostaining endogenous APC using anti-APC antibody was performed. Enlarged images show representative axonal growth cones. Note that the RFP-KIF5C dominant negative constructs hardly reach the growth cones. Asterisks indicate cell bodies. Scale bar 50 μm. (**b**) Graph showing APC fluorescence intensity levels in growth cones under the indicated conditions. Shown are means  +/− SEM from 3 independent experiments; RFP (n = 18 neurons), RFP-KIF5C355 (n = 22 neurons) and RFP-KIF5C678 (n = 20 neurons). An independent sample t-test was used for comparison. **p < 0.01.

**Figure 7:**
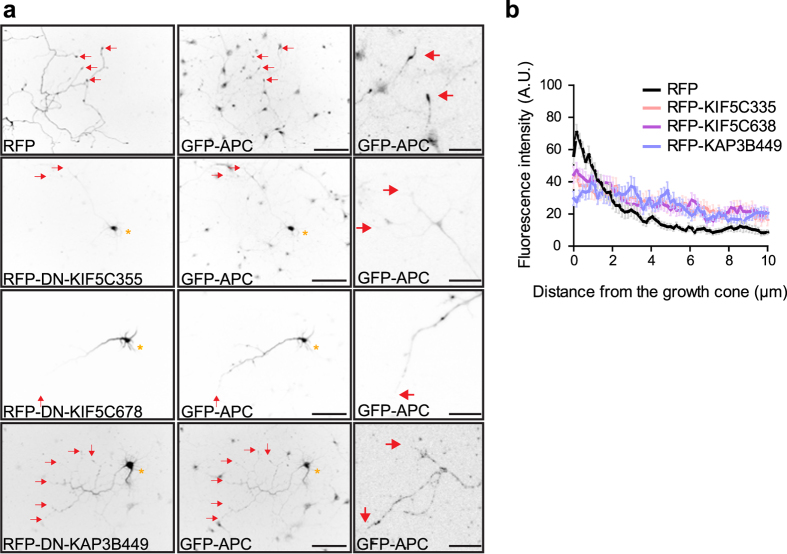
Dominant negative kinesin-1 and kinesin-2 constructs prevent the distal axonal enrichment of GFP-APC. (**a**) E18 rat hippocampal neurons co-transfected at 1 DIV with GFP-APC and RFP, RFP-KIF5C355, RFP-KIF5C678 or RFP-KAP3B449 constructs and fixed at 4 DIV. PFA fixation and immunostaining using anti-GFP antibody was performed. Asterisks indicate cell bodies and arrows indicate axonal tips or growth cones. Scale bar 100 μm. Scale bar in enlarged images, 20 μm. (**b**) Linescan plots showing the fluorescence intensity distribution of GFP-APC along the axon when co-expressed with RFP, RFP-KIF5C355, RFP-KIF5C678 or RFP-KAP3B449. Shown are means  +/− SEM from 3 independent experiments, n = 14 and n = 27, n = 37 and n = 38, respectively.

To confirm that kinesin-1 function is required for APC enrichment at the axon tip we used KIF5 fragments lacking the motor domain (Fig. 6a), which have been shown to act dominant negatively and inhibit axonal trafficking of kinesin-1 cargo, such as Kv1 K(+) channels and GABA type B receptors^54^,^55^,^56^. Expression of RFP-KIF5C335 or RFP-KIF5C678 significantly reduced the level of endogenous APC in the growth cones of neurons (Fig. 6b,c). In addition the axon tip enrichment of GFP-APC was abolished by both these kinesin-1 DN constructs, as shown quantitatively by fluorescence intensity linescans (Fig. 7).

In non-neuronal cells, heterotrimeric kinesin-2 is the predominant motor driving peripheral localisation of APC (Fig. 3, and ref. 6). Moreover, in mouse DRG neurons an N-terminal fragment of APC containing the kinesin-2 binding domain accumulates at axon tips^34^. To assess how heterotrimeric kinesin-2 contributes to axon tip localisation in neurons, a KAP3 dominant negative fragment previously used to inhibit peripheral accumulation of APC in epithelial cells^6^, RFP-KAP3B449, was expressed in neurons. Interestingly, RFP-KAP3B449 also inhibited axon tip enrichment of GFP-APC (Fig. 7), suggesting that APC accumulates at the axon tip through interactions with both kinesin-1 and kinesin-2.

### The kinesin-1 binding domain is required for distal axonal enrichment of the APC C-terminus

**Figure 8:**
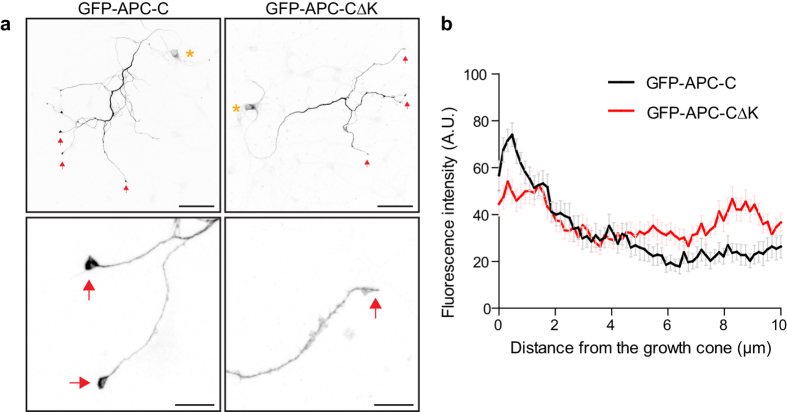
Kinesin-1 binding is required for the distal axonal enrichment of the C-terminus of APC, which lacks the kinesin-2 interacting domain. (**a**) E18 rat hippocampal neurons expressing GFP-APC-C or GFP-APC-CdK constructs and fixed with PFA at 4 DIV. Asterisks indicate cell bodies and arrows indicate axonal tips or growth cones. Scale bar 100 μm. Scale bar in enlarged images, 20 μm. (**b**) Linescan plots showing the fluorescence intensity distribution of GFP-APC-C or GFP-APC-CdK along the axon. Shown are means  +/− SEM from 3 independent experiments, n = 24 and n = 30, respectively.

The C-terminus of APC, containing the MT- and EB-binding domains but lacking the kinesin-2 binding region, has been shown to be enriched at axon tips when expressed in cortical neurons from conditional APC knockout mice and in DRG neurons from wild type mice^30^,^34^. Similarly, we found that GFP-APC-C accumulated at axonal tips when expressed in rat hippocampal neurons; strikingly, however, GFP-APC-CΔK did not (Fig. 8). These data demonstrate that an interaction with EB1 is not sufficient to concentrate APC-C at axon tips, since GFP-APC-CΔK could still bind and colocalise with EB1 (Fig. 2), and suggest that the interaction with kinesin-1 is required for axon tip accumulation of APC-C. Since the APC N-terminus was observed to accumulate at axon tips^34^, it would appear that APC fragments can be efficiently transported along axons by individual kinesins whereas full length APC requires the combined activity of both kinesin-2 and kinesin-1 through interactions at the N-terminus and C-terminus, respectively.

## Discussion

APC is a central player in cell polarisation and axon growth through its interactions with the cytoskeleton^3^. In non-neuronal cells APC is known to accumulate peripherally through the action of kinesin-2 MT motor proteins and EB +TIPs^4^,^5^,^6^. Here we have shown that kinesin-1 heavy chains bind directly to the C-terminus of APC, between the MT- and EB-binding regions, and that through this interaction kinesin-1 functions alongside heterotrimeric kinesin-2 to localise GFP-APC distally in fibroblasts and neurons.

Characterisation of kinesin-1 as a novel binding partner of APC has implications for the tumour suppressor activity of APC, since the interaction occurs through the C-terminal region that is lost after tumourigenic truncating mutations^17^. Reductions in β-catenin destruction complex efficiency, the loss of MT association and ablation of EB1 binding after APC truncation have all been linked with gut epithelium transformation^57^. We have shown that deletion of the kinesin-1 binding region attenuates the peripheral localisation of APC in fibroblasts. This interaction could also function to regulate APC localisation in epithelia, perhaps contributing to the polarisation and regeneration pathways which become disrupted after APC truncation. Concomitant roles for kinesins-1, -2, and EB1 are suggestive of tight controls on APC distribution, perhaps enabling differential localisation of distinct APC complexes through interactions at either the N- (kinesin-2) or C-terminus (kinesin-1, EB1)^13^.

In neurons, APC functions in axon specification, growth and dynamics, and is distally localised to the axon tip by a previously poorly understood mechanism. Kinesin-1 is a crucial motor in axonal cargo delivery, functioning in bulk trafficking of axonal material through both fast membrane transport and slow cytoplasmic protein transport^42^,^58^. Our finding that APC lacking the kinesin-1 binding region fails to accumulate at the axon tip in rat hippocampal neurons strongly implicates direct binding to kinesin-1 motor subunits as a requirement for axon tip accumulation. Similar direct coupling to KIF5 chains has been observed for a number of axonal and dendritic cargo molecules, such as the multi-receptor adaptor GRIP1, the mitochondrial adaptors syntaphilin and Milton/TRAK, and the synaptic vesicle adaptor syntabulin^59^,^60^,^61^,^62^,^63^,^64^. Interestingly, APC has recently been implicated in mitochondrial positioning, and is proposed to be recruited to mitochondria via an interaction between its C-terminal domain and the Milton/Miro complex^65^. This interaction is likely mediated by KIF5, since it binds directly to both APC (this work) and Milton^63^,^64^.

Intriguingly, both kinesin-1 and kinesin-2 activity is required for neuronal APC localisation, since APC axon tip accumulation was also disrupted by expression of DN fragments of the KAP3 subunit of heterotrimeric kinesin-2 (ref. 6; Fig. 7). Overexpression of the DN APC-binding fragment of KAP3 may also have blocked other known interactors with the APC N-terminal armadillo region, which could have contributed to the localisation phenotype observed^14^,^66^. The requirement of N-terminal interactions for proper localisation further suggests that APC trafficking is tightly controlled, while the activity of two kinesins in this process sets APC apart from other axonal cargos. The major difference for cargo transport between most non-neuronal cells and a neuronal axon is the distance that needs to be travelled, which could be up to a metre. Axonal transport therefore has to be tightly regulated but also capable of adapting to the changing axonal environment to overcome roadblocks.

The two kinesins could be required for distinct stages in APC axonal trafficking. For example, an increased proportion of GTP-tubulin and detyrosinated MTs in the axon initial segment and axon shaft has been found to favour kinesin-1 motility, resulting in biased axonal trafficking of kinesin-1 cargo^37^,^67^,^68^,^69^. Moreover, dynamic, tyrosinated MTs, which are the preferred substrate of kinesin-2 (ref. 70), are enriched at axon tips^31^,^71^,^72^. Indeed, the heterotrimeric kinesin-2 subunit KIF3C has recently been characterised as an axon tip-localised kinesin, preferentially associating with tyrosinated microtubules and functioning with EB proteins to regulate axon growth^73^. One possibility is that coupling to kinesin-1 provides efficient transport of APC through the axon initial segment and along the axon shaft before handing over to kinesin-2 and EB proteins for enrichment at the axon tip. Arguing against this simple model is the observation that both the N- and C-terminal fragments of APC accumulate in the distal axon (ref. 34; this study), despite interacting with only kinesin-2 or kinesin-1, respectively. This raises the possibility that non-sequential interactions with kinesins-1 and -2 are required for targeting APC to the axon tip. However, it is also conceivable that only full length APC requires the hand-over from kinesin-1 to kinesin-2. There is also potential for kinesin-specific roles in transporting distinct APC complexes in neurons^13^.

Our finding that specific disruption of kinesin-1 binding to APC leads to decreased axon growth highlights the importance of proper localisation of APC to neuronal development. These results are consistent with experiments using DN APC constructs which demonstrate that APC and its binding partners play roles in axon specification and growth^28^,^34^. APC may not be absolutely required for these processes, however, since knock out experiments in Drosophila and mouse yielded polarised neurons with wild type-like axon length^30^,^66^, although the mouse neurons exhibited highly disorganised MTs and actin in oversized, often split, growth cones^30^. Interestingly, we did not observe any growth cone phenotypes in neurons expressing GFP-APCΔK (data not shown). Sequestration of binding partners by APC DN constructs has been proposed to underlie the difference in outcomes between knock out and DN experiments^66^. The GFP-APCΔK mutant analysed here could therefore be having DN effects, disrupting interactions between APC and its binding partners leading to shorter axons.

To understand the complex function and pathology of APC in neuronal and other tissues we need to understand fully the interplay between APC and its many interactors. The finding that the APC protein uses two distinct processive kinesins for its enrichment in the distal axon provides new insight into how the specific distribution of APC complexes is achieved.

## Materials and Methods

### Rat hippocampal neuron dissociation, culture and transfection

Primary hippocampal cultures were prepared from embryonic day 18 (E18) rat brains^74^. Cells were plated in 12 well plates on coverslips coated with poly-L-lysine (30 μg/ml) and laminin (2 μg/ml) at a density of 75,000/well. Hippocampal cultures were grown in Neurobasal medium (NB) supplemented with B27, 0.5 mM glutamine, 12.5 μM glutamate and penicillin/streptomycin. One day after plating, hippocampal neurons were transfected using Lipofectamine 2000 (Invitrogen). Briefly, DNA (3.6 μg/well) was mixed with 3 μl Lipofectamine 2000 in 200 μl NB, incubated for 30 minutes and then added to the neurons in NB at 37 °C in 5% CO2 for 45 min. Next, neurons were washed with NB then incubated in the original medium at 37 °C in 5% CO2 for 3 days.

### Non-neuronal cell culture and transfection

HeLaM (Dr A. Peden, University of Sheffield, UK) and MRC-5 (ECACC) cells were cultured in DMEM supplemented with 10% FCS and 2 mM glutamine at 37 °C in a humidified, 8% CO_2_ environment. Cells were transfected with DNA by addition of DNA constructs together with JetPEI (QBiogene), using half the manufacturer’s recommended amounts, followed by incubation in culture conditions for 16–24 hours. Cells were transfected with siRNA using ONTARGETplus SMARTpool siRNA duplexes (Dharmacon) together with INTERFERIN (QBiogene), according to the manufacturer’s instructions. siRNA-transfected cells were incubated for 72 hours prior to fixation, with DNA transfection carried out in fresh media after 48 hours if necessary.

### Expression constructs

GFP-APC was a kind gift from Inke Näthke (University of Dundee, UK). GFP-APC deletion constructs were generated by PCR from GFP-APC and cloned into pEGFP-C1/C2 (Clontech). GST-APC deletion constructs were generated by PCR using GFP-APC and cloned into pGEX-4T-1 (GE Healthcare). GST-KIF5Bct, GST-KLC1Bct and GST-KLC2ct were previously described^75^. GFP-KIF5Bct and 6His-KIF5Bct were subcloned from GST-KIF5Bct into pEGFP-C1 and pRSETA (Invitrogen), respectively. GST-KAP3Bct (amino acids 512–793) was amplified with specific primers and cloned into the pGEX bacterial expression vector. RFP-KAP3B449 (amino acids 449–793) was generated by PCR from KAP3B-myc and cloned into pcDNA3.1(+)-RFP. KIF5A-myc/his and KIF5C-myc/his were kind gifts from Gerado Morfini (University of Illinois, US). GST-KIF5Act (amino acids 774–1032) and GST-KIF5Cct (amino acids 773–956) were made by PCR from KIF5A-myc/his and KIF5C-myc/his, respectively, and cloned into pGEX-4T-1. RFP-KIF5C335 (amino acids 335–956) and RFP-KIF5C678 (amino acids 678–956) were generated by PCR from KIF5C-myc/his. GST-EB1 was a kind gift from Anne Straube (University of Warwick).

### Antibodies

Antibodies were obtained from the following sources: anti-APC (ALi12-28, Abcam; C20, Santa Cruz), anti-KHC (Ron Vale, University of California, San Francisco), 1A11/4 anti-EB1 (Cell Signalling Technology), TAT-1 anti-α-tubulin for western blotting (Keith Gull, University of Oxford), YL1/2 anti-tyrosinated α-tubulin for immunofluorescence (Millipore), 6-11B-1 anti-acetylated α-tubulin (Sigma), anti-KAP3 (Epitomics), anti-GFP antibody for western blotting (Roche), anti-GFP for immunofluorescence in MRC-5 cells (Phillip Woodman, University of Manchester), anti-GFP for immunofluorescence in neurons (Abcam), HIS-1 anti-polyHistidine (Sigma-Aldrich), fluorescently-labelled secondary antibodies for immunofluorescence (Jackson ImmunoResearch Laboratories, Stratech Scientific or Invitrogen) and IRDye 700CW- and 800C-labelled secondary antibodies for western blotting (LI-COR Biosciences).

### Hippocampal Neuron Immunofluorescence

Hippocampal neurons on glass coverslips were fixed for 10 minutes in 4% paraformaldehyde at room temperature, permeabilised for 15 minutes at room temperature in PBS containing 0.1% Triton X-100, then labelled with primary antibodies in PBS and 10% goat serum for 1 hour and secondary antibodies in PBS for 1 hour. PBS washes were performed after each antibody incubation. Coverslips were mounted on glass slides in Fluorsave (Calbiochem). Samples were imaged using an upright Nikon Eclipse 80i microscope with a 20 × 0.75 N.A. Plan Fluor objective, a 40 × 1.3 N.A. Plan Fluor objective, or a 60 × 1.4 N.A Plan Apo VC objective and images were captured with a Photometrics CoolSNAP HQ2 CCD camera, using the Nikon NIS Br software. All images were scaled and prepared in ImageJ and Adobe IllustratorCS.

### Non-neuronal Cell Immunofluorescence

MRC-5 cells grown on glass coverslips were fixed for 5 minutes in methanol at −20 °C. Fixed cells were labelled with primary antibodies in PBS for 1 hour and secondary antibodies in PBS for 30 minutes. PBS washes were performed after each antibody incubation and 0.1 μg/ml 4′,6-diamidino-2-phenylindole (DAPI) was added to the final wash to stain nuclei. Coverslips were mounted on glass slides in Mowiol containing 25 mg/ml 1,4-diazabicyclo[2.2.2]octane or Prolong Diamond (Invitrogen).

Widefield images were obtained using an Olympus BX-60 microscope with a 60 × 1.40 N.A. UPlanApo objective and a CoolSnap ES camera, using MetaVue software (Molecular Devices). Confocal images were collected on a Leica SP8 inverted confocal using an HC PLAPO CS2 100x/1.40 NA oil objective with variable confocal zoom. The confocal settings were as follows: pinhole 1 airy unit; scan speed 400Hz unidirectional with a 3 line average; format 1024 × 1024 or 2048 × 2048. Images were collected using the following settings: GFP 493–538 nm, hybrid detector; Cy3 558–605 nm, hybrid detector; Cy5 657–730 nm, PMT detector, using the white light laser set at 484 nm (~23%), 550 nm (~24%) and 647 nm (~26%) intensities, respectively. To eliminate cross-talk between channels, the GFP and C5 images were collected simultaneously, followed by the Cy3 image. When acquiring 3D optical stacks the confocal software was used to determine the optimal number of Z sections, which was then doubled. Only the maximum intensity projections of these 3D stacks are shown in the results, using all relevant z-planes for the full images and three z-planes for zoomed areas. All images were scaled and prepared into figures using FIJI^76^ and Adobe IllustratorCS. In order to make fainter structures visible for display purposes, the brightness and contrast of some images have been adjusted so that certain areas are saturated.

### Recombinant protein expression

Recombinant protein expression was induced in BL21 bacteria, grown with agitation at 37 °C to OD_600 _= 0.6, by addition of 500 μM IPTG. After induction for 3 hours in growth conditions, bacteria were collected by centrifugation and lysed in cold lysis buffer (50 mM Tris pH 7.4, 150 mM NaCl, 1% Triton X-100, protease inhibitor cocktail). This recombinant protein expression lysate was sonicated and insoluble material cleared by centrifugation (14,000 × g) for 30 minutes before a sample was purified using glutathione- or Ni-sepharose to assess expression level.

### GST pull-down assays

Cells grown in 10 cm dishes were lysed in 600 μl cold lysis buffer for 10 minutes prior to centrifugation at 14,000 × g for 5 minutes. The supernatant was collected and 300 μl was isolated for addition of normalised amounts of recombinant GST-tagged protein expression lysate. This mixture was incubated on a rotator at 4 °C for 2 hours prior to addition of glutathione sepharose and incubation for a further hour. Sepharose beads were collected by centrifugation (300 × g), washed 3 times in cold lysis buffer and prepared for SDS PAGE by the addition of sample buffer. For GST pull-downs of recombinant 6His-tagged proteins, normalised amounts of GST- and 6His-tagged protein expression lysate was added to 500μl cold blocking solution (lysis buffer containing 2% w/v skimmed milk, clarified by centrifugation at 13,000 rpm in a microfuge). These pull-downs were then treated as described for cell lysate GST pull-downs.

### SDS PAGE and Western Blotting

Protein samples were separated by SDS PAGE using lab-made 8%, 10%, 14%, or pre-cast 4–15% gradient acrylamide gels (Biorad), and transferred onto nitrocellulose membrane. Protein transferred to the membrane was visualised by Ponceau-S staining and subsequently destained in PBS. Membranes were then incubated for 30 minutes with agitation in blocking buffer (4% w/v skimmed milk, PBS). Incubation with primary antibody for 2 hours in blocking buffer containing 0.5% tween was followed by secondary antibody incubation for 1 hour in 0.5% tween blocking buffer. Membranes were washed in PBS containing 0.5% tween after each antibody incubation and analysed using the LI-COR Odyssey infrared imaging system (LI-COR Biosciences).

### Quantification of axonal length

ImageJ software^77^ was used to analyse the images. The drawing tool was used to trace and measure the length of the primary axonal neurite per neuron from the axon hillock to the growth cone.

### Quantification of fluorescence intensity line scans

All images were taken at the same settings for light and exposure with parameters adjusted so that the pixel intensities were below saturation. Images were analysed using ImageJ analysis software. For quantification of fluorescence intensity levels and distribution, the length of an axon was traced using the tip of the growth cone as the starting point. The intensity of the background was similarly measured in areas adjacent to the axons and subtracted from the axon value giving a final intensity value per axon.

### Quantification of growth cone immunofluorescence

All images were acquired and analysed as described above. For quantification of intensity levels of tyrosinated and acetylated microtubules and APC, the whole area of a growth cone was traced using Image J software and the average pixel intensity per unit area was calculated. The intensity of the background was similarly measured in areas adjacent to the growth cone and subtracted from the growth cone value giving a final intensity value per growth cone. For each neuron the fluorescence intensity of several growth cones was averaged to give a final intensity value per neuron.

## Additional Information

**How to cite this article**: Ruane, P. T. *et al*. Tumour Suppressor Adenomatous Polyposis Coli (APC) localisation is regulated by both Kinesin-1 and Kinesin-2. *Sci. Rep.* **6**, 27456; doi: 10.1038/srep27456 (2016).

## Supplementary Material

Supplementary Information
