# Supplementary material for: Tumour Suppressor Adenomatous Polyposis Coli (APC) localisation is regulated by both Kinesin-1 and Kinesin-2
Source: Sci Rep. 2016 Jun 7;6:27456. doi: 10.1038/srep27456 (PMC4895226; doi:10.1038/srep27456)
Supplement: Supplementary Information [file srep27456-s1.pdf]

Tumour Suppressor Adenomatous Polyposis Coli (APC) localisation is regulated by both Kinesin-1 and Kinesin-2

Peter Ruane, Laura F. Gumy, Becky Bola, Beverley Anderson, Marcin J. Wozniak, Casper C. Hoogenraad and Victoria J. Allan

Supplementary material

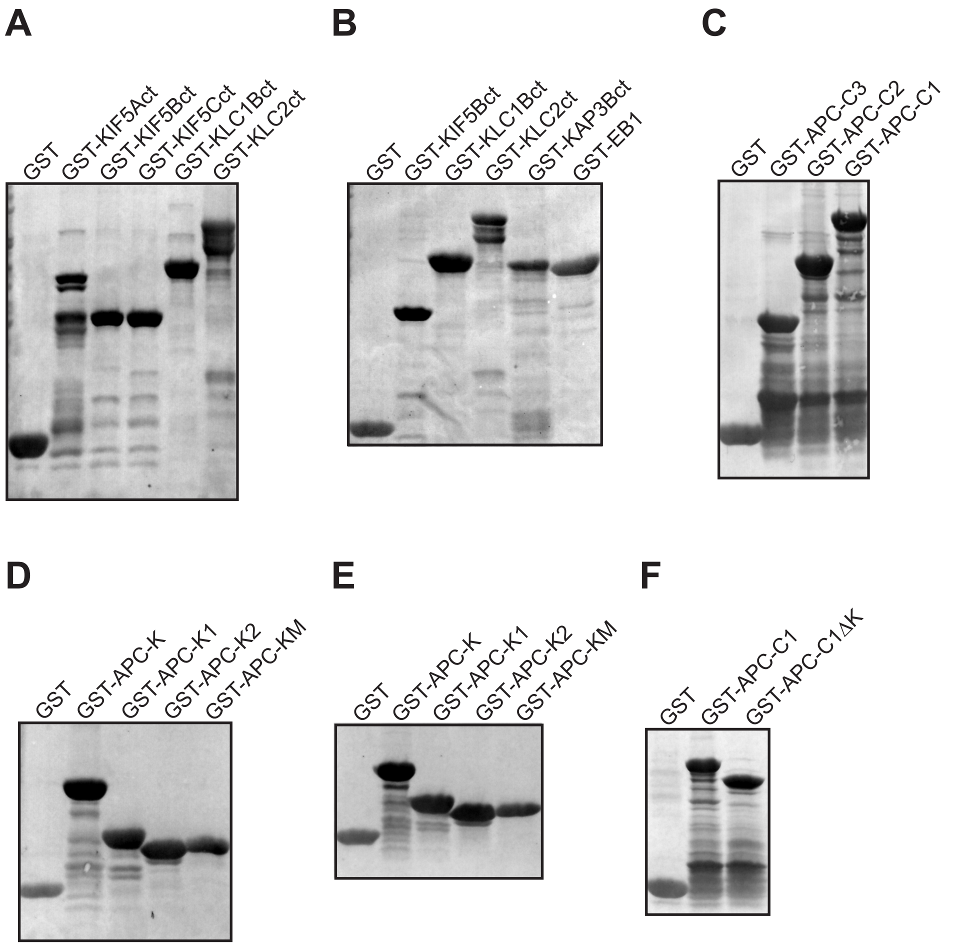

Supplemental Figure 1. Images of Ponceau-S-stained membranes used for the immunoblotting analysis shown in Figure 1. The panel order is the same in both Figures.

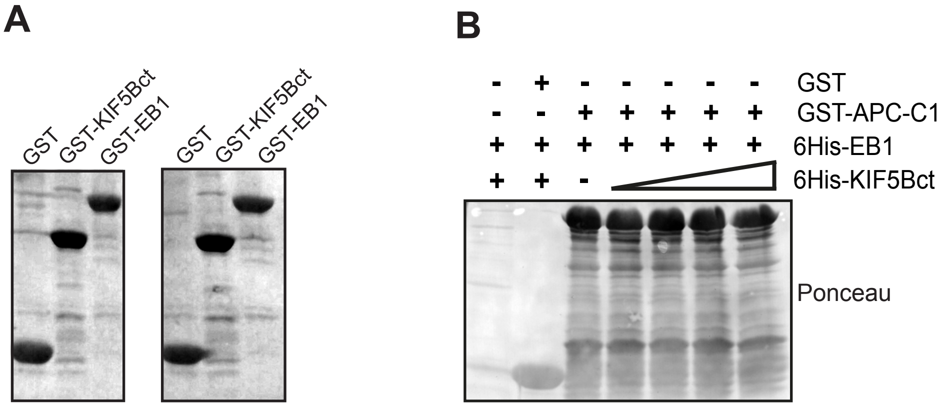

Supplemental Figure 2. Ponceau-S staining of membranes used for the immunoblotting analysis shown in Figure 2. (**A**) Ponceau-S staining for Figure 2A. (**B**) Ponceau-S staining for Figure 2D.

Figure S3

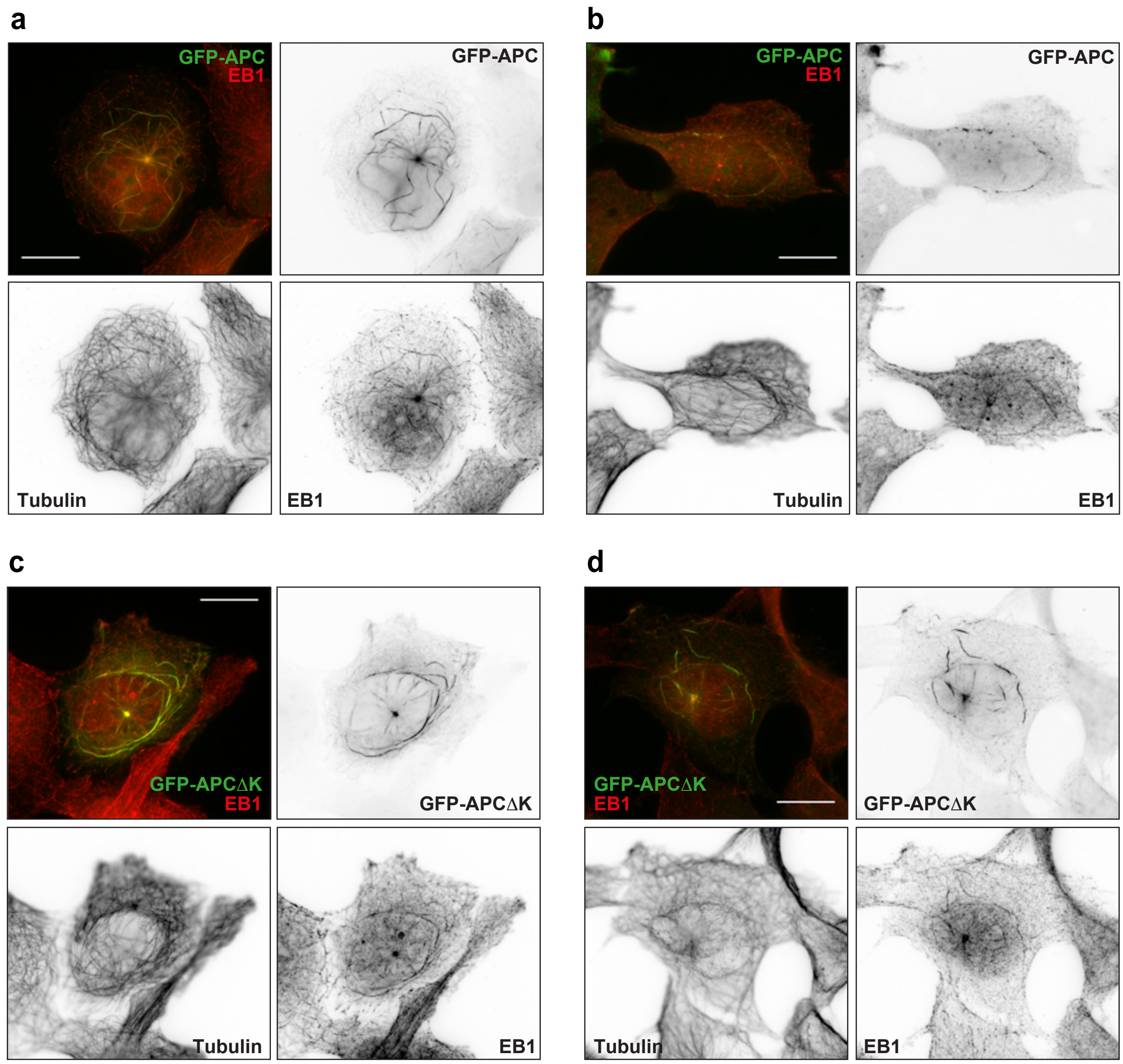

Supplemental Figure 3. Wide-field images of MRC-5 cells where GFP-APC and GFP-APCΔK were not peripherally localised. Cells transfected with full length GFP-tagged APC with (A, B: GFP-APC) or without (C, D: GFP-APCΔK) the KIF5 binding region were labelled with anti-EB1 (red) and anti-α-tubulin. Merges of the GFP and EB1 channels are shown, and single channels are presented in reverse contrast. Scale bars, 10μm.
